# Apparent Histologic Discordance in Recurrent Testicular Germ Cell Tumor With Bulky Retroperitoneal Disease and Inferior Vena Cava Compression: A Case Report and Brief Review of the Literature

**DOI:** 10.7759/cureus.116587

**Published:** 2026-09-20

**Authors:** Alireza Izadian Bidgoli, Alberto Gomez Veliz, Nikunj Shah, Hellen Ailette Gomez Usatorres, Alberto Gomez Usatorres

**Affiliations:** 1 General Surgery, American University of the Caribbean School of Medicine, Cupecoy, SXM; 2 Internal Medicine, Jackson Memorial Hospital, Miami, USA; 3 Pathology, University of Miami/Jackson Health System, Miami, USA; 4 Internal Medicine, Universidad Hispanoamericana, San José, CRI

**Keywords:** inferior vena cava, neoplasm recurrence, retroperitoneal neoplasms, seminoma, testicular neoplasms

## Abstract

Testicular germ cell tumors are highly curable, but bulky retroperitoneal recurrence creates diagnostic uncertainty when a limited biopsy does not capture all components of the primary tumor. A 35-year-old man presented with abdominal pain radiating to the back and intermittent constipation nearly five years after an orchiectomy for a 6.5-cm mixed germ cell tumor consisting of seminoma (80%) and embryonal carcinoma (20%) with lymphovascular invasion. CT showed a heterogeneous 14.1-cm retroperitoneal nodal mass with near-complete inferior vena cava effacement. Retrospective comparison with prior imaging identified a 2.1-cm focus at the same site on a June 2022 study; no intervening imaging was available. Lactate dehydrogenase (LDH) was 630 U/L (2.25 times the upper limit of normal), beta-human chorionic gonadotropin (β-hCG) was minimally elevated, and alpha-fetoprotein (AFP) was normal. A core biopsy showed seminoma with focal necrosis; no nonseminomatous component was identified in the sampled tissue.

With the pulmonary micronodules regarded as indeterminate rather than metastatic, the available data were consistent with cN3 cM0 S2, stage IIIB. Because the primary tumor contained embryonal carcinoma and because core sampling could not exclude unsampled nonseminoma, the oncology team applied an intermediate-risk nonseminomatous germ cell tumor framework and initiated curative-intent bleomycin, etoposide, and cisplatin, with four cycles planned. At the last available follow-up, the patient had completed the inpatient portion of cycle 1 without major acute toxicity and reported improved pain; neither treatment completion nor radiographic response was available. Apparent histologic discordance on core biopsy should be interpreted in light of the original pathology, serum markers, imaging, and multidisciplinary assessment.

## Introduction

Testicular germ cell tumors are classified as seminomatous or nonseminomatous, and finding any nonseminomatous component in a mixed tumor has important implications for staging and treatment [1-3]. After orchiectomy for clinical stage I nonseminoma, accepted management options include surveillance, risk-adapted adjuvant chemotherapy, and retroperitoneal lymph-node dissection in selected patients [3,4]. Lymphovascular invasion is the best-established predictor of relapse, so adherence to scheduled clinical assessment, serum tumor-marker testing, and cross-sectional imaging is particularly important [3,5,6].

Testicular germ cell tumors commonly spread through the retroperitoneal lymphatic system, where progressive nodal disease may cause abdominal or back pain, gastrointestinal symptoms, or compression of major vessels [1,6]. Mixed germ cell tumors may be spatially heterogeneous, and a core biopsy from a large recurrent mass may not capture every histologic component [2,7,8]. We present a case of bulky retroperitoneal recurrence with near-complete inferior vena cava effacement after orchiectomy for a mixed seminoma-embryonal carcinoma germ cell tumor, in which a limited core biopsy of the recurrent site showed only seminoma. This report examines the interpretation of apparent histologic discordance and the multidisciplinary management of vessel-involving retroperitoneal recurrence.

## Case presentation

Clinical presentation

A 35-year-old Hispanic man of Cuban nationality with a history of a right-sided testicular germ-cell tumor presented to the emergency department on July 31, 2026, with a one-day history of lower abdominal pain radiating to his back and intermittent constipation. He denied fever, chills, nausea, vomiting, diarrhea, chest pain, dyspnea, or urinary symptoms. His medical history included depression, Ehlers-Danlos syndrome, and class III obesity. He denied tobacco use, alcohol use, vaping, and recreational drug use.

The patient had undergone a right radical orchiectomy on August 30, 2021. The resected tumor measured 6.5 cm and consisted of seminoma (80%) and embryonal carcinoma (20%). Lymphovascular invasion and tumor necrosis were present, but the tumor was confined to the testis, and surgical margins were negative. Germ-cell neoplasia in situ was not identified. The primary tumor was classified as pT2; regional lymph nodes were not assessed pathologically. Preoperative tumor markers included a lactate dehydrogenase (LDH) level of 1,479 U/L, an alpha-fetoprotein (AFP) level of 4.9 ng/mL, and a negative beta-human chorionic gonadotropin (β-hCG) result. Initial staging imaging reportedly showed no definite metastatic disease. He did not undergo retroperitoneal lymph node dissection or receive adjuvant chemotherapy.

On retrospective comparison of the CT examination from July 2026 with the June 4, 2022 study, the radiologist identified a 2.1-cm retroperitoneal focus at the site of the subsequent mass. The 2026 report described the 2022 examination as showing fatty liver and no acute findings. The available records did not indicate that the focus had been recognized as suspicious for recurrence at that time, that oncologic reassessment or short-interval imaging had been recommended, or that additional cross-sectional imaging had been performed before the 2026 presentation. Consequently, the reason for the absence of interval evaluation cannot be established from the available record.

Initial assessment and physical exam

At presentation, the temperature was 37.0°C, the heart rate was 106 beats/min, the respiratory rate was 16 breaths/min, the blood pressure was 156/115 mmHg, and the oxygen saturation was 95% on room air. The patient was alert and in no acute distress. Cardiopulmonary examination revealed a regular rhythm and clear breath sounds. The abdomen was soft and nondistended, with mild lower-quadrant tenderness but no guarding or rebound tenderness. After transfer for oncologic evaluation, he remained hemodynamically stable. The left testis was normal on examination, and the prior right inguinal surgical site was well healed. No palpable lymphadenopathy or lower-extremity edema was identified, despite the inferior vena cava (IVC) compression that was demonstrated later.

Laboratory and diagnostic testing

Initial laboratory testing revealed a white blood cell count of 12.9 × 10³/µL, a hemoglobin level of 14.1 g/dL, and a platelet count of 306 × 10³/µL. Serum creatinine was 0.60 mg/dL. Liver chemistry testing showed an aspartate aminotransferase (AST) level of 117 U/L, an alanine aminotransferase (ALT) level of 211 U/L, and an alkaline phosphatase level of 142 U/L. Serum bilirubin and albumin were within the reference ranges reported. Tumor markers included an LDH level of 630 U/L, a β-hCG level of 5.2 mIU/mL, and an AFP level ranging from 1.2 to 1.8 ng/mL. A later β-hCG measurement was 4.5 mIU/mL.

Among the later inpatient measurements available for review, the creatinine level ranged from 0.50 to 0.60 mg/dL, the AST level from 25 to 105 U/L, and the ALT level from 74 to 206 U/L. LDH was 569 U/L on August 9, 429 U/L on August 11, 371 U/L on August 13, and 321 U/L on August 14. Table 1 summarizes selected admission values, the observed inpatient ranges, and measurements obtained around discharge.

**Table 1:** Laboratory findings at admission, during hospitalization, and at discharge The hospitalization column reports the observed minimum-to-maximum range among subsequent inpatient measurements. The discharge column summarizes available measurements obtained during the discharge interval; when two measurements were available, they are presented as a range. Reference ranges are those of the reporting laboratory AFP: alpha-fetoprotein; ALT: alanine aminotransferase; AST: aspartate aminotransferase; β-hCG: beta-human chorionic gonadotropin; LDH: lactate dehydrogenase; NR: not reported

Laboratory parameter	Admission	Range during hospitalization	At discharge	Reference range	Unit
White blood cell count	12.9	7.5–13.7	7.5–13.7	4.5–11.0	×10³/µL
Hemoglobin	14.1	12.7–14.1	12.7–13.4	13.5–17.5	g/dL
Platelet count	306	318–365	318–330	150–400	×10³/µL
Creatinine	0.60	0.50–0.60	0.50–0.60	0.70–1.30	mg/dL
AST	117	25–105	25–45	10–40	U/L
ALT	211	74–206	74–119	7–56	U/L
LDH	630	321–569	321	140–280	U/L
β-hCG	5.2	4.5	NR	< 5.0	mIU/mL
AFP	1.8	NR	NR	< 10.0	ng/mL

Contrast-enhanced CT of the abdomen and pelvis revealed a heterogeneous retroperitoneal mass measuring approximately 9.5 × 8.6 cm in the axial plane and 14.1 cm in craniocaudal length. The mass extended toward the pelvic inlet and caused marked compression and near-complete effacement of the IVC, with substantial mass effect on adjacent retroperitoneal structures and bowel (Figure 1). Comparison with the earlier examination showed enlargement at the site of the retrospectively identified 2.1-cm focus.

**Figure 1:**
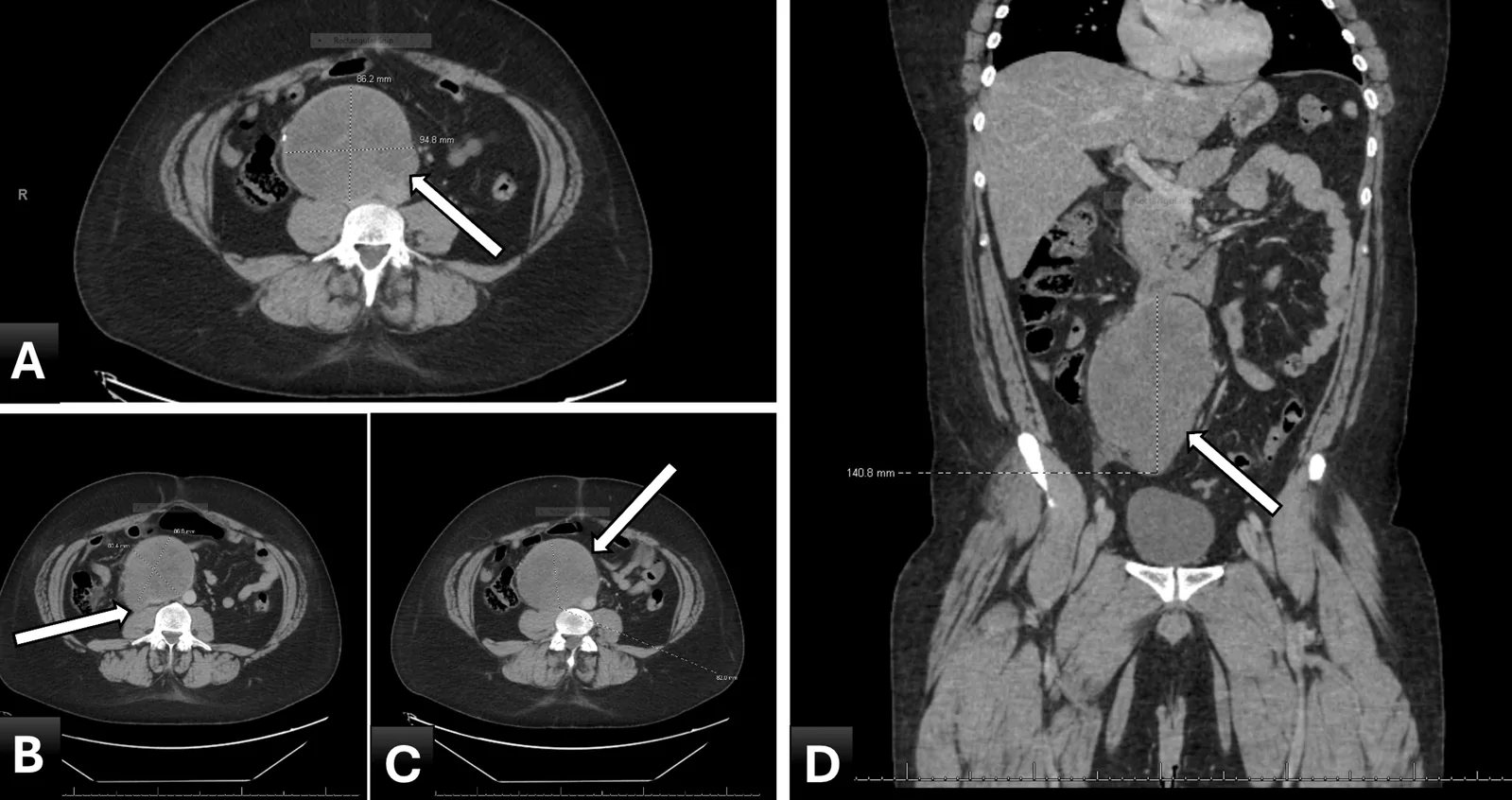
Bulky retroperitoneal nodal recurrence with inferior vena cava compression Axial and coronal contrast-enhanced CT images demonstrate a large heterogeneous retroperitoneal mass. The lesion measures approximately 9.5 × 8.6 cm axially (A) and extends approximately 14.1 cm craniocaudally to the pelvic inlet (D). The mass produces marked compression and near-complete effacement of the inferior vena cava (B, arrow) and substantial mass effect on surrounding structures and adjacent bowel (C). Comparison with prior imaging showed marked enlargement from a previously documented 2.1-cm retroperitoneal nodal focus. Core biopsy demonstrated seminoma in the sampled tissue CT: computed tomography

Staging CT of the chest revealed three nonspecific noncalcified bilateral pulmonary micronodules measuring up to 3 mm and a subcentimeter right hilar lymph node, with no pathologic thoracic lymphadenopathy and no definite pulmonary metastases. A 2.8-cm triangular anterior mediastinal soft-tissue density that contained interdigitating fat was more consistent with residual thymic tissue or thymic hyperplasia. Hepatic steatosis was also seen in the visualized upper abdomen (Figure 2).

**Figure 2:**
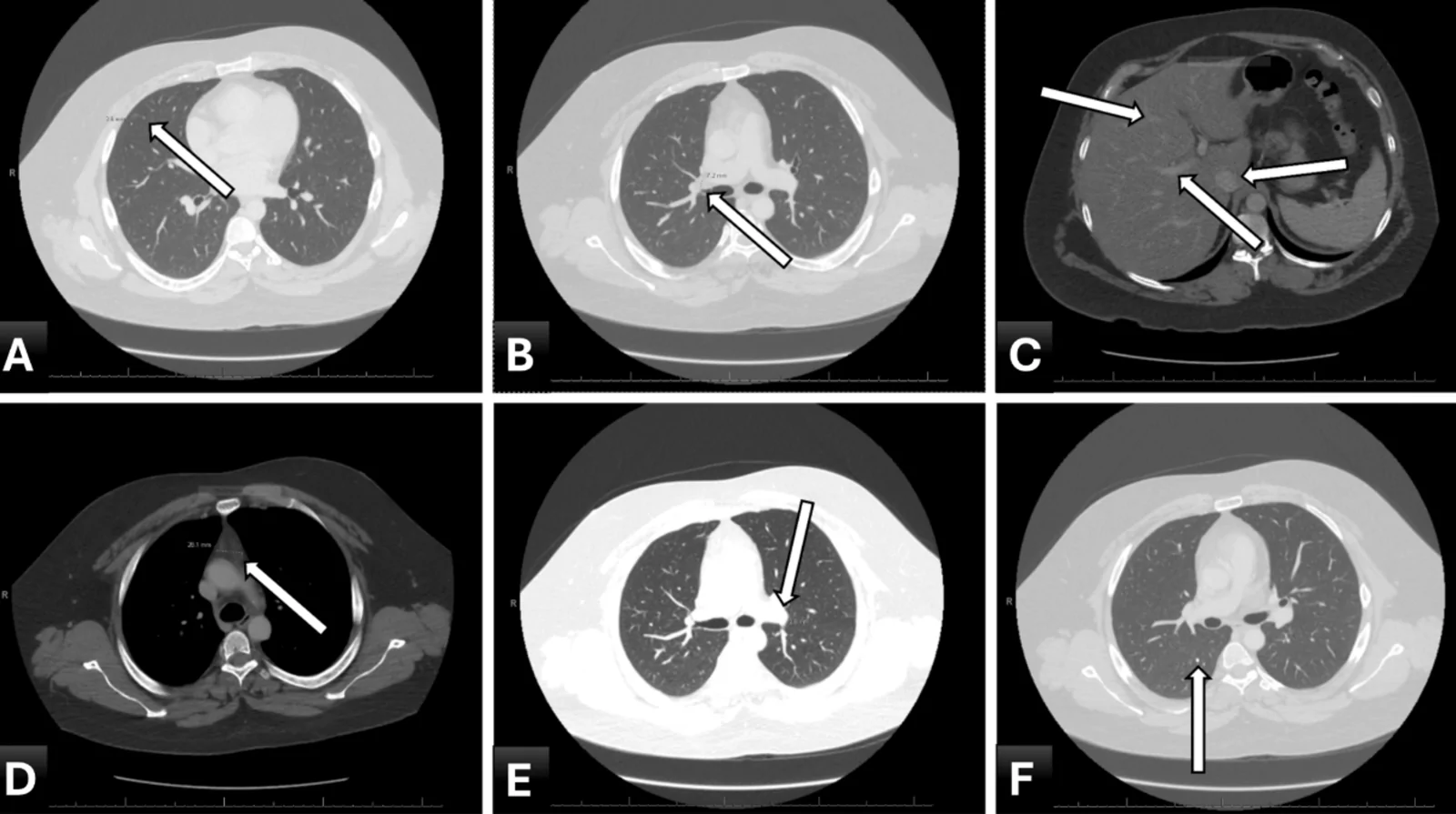
Thoracic staging CT findings in recurrent testicular germ-cell tumor Axial contrast-enhanced CT images demonstrate a 2.8-mm noncalcified right middle-lobe pulmonary nodule (A), a 7.2-mm right hilar lymph node that does not meet pathologic size criteria (B), and diffuse hepatic hypoattenuation consistent with hepatic steatosis in the visualized upper abdomen (C). A 2.8-cm triangular anterior mediastinal soft-tissue density containing interdigitating fat was favored to represent residual thymic tissue or thymic hyperplasia (D). Additional approximately 3-mm perifissural nodules are present in the left upper lobe (E) and left lower lobe (F). The pulmonary micronodules were nonspecific, with no definite thoracic metastatic disease or pathologically enlarged thoracic nodes CT: computed tomography

Because the radiographic appearance was highly concerning for recurrent germ-cell malignancy, the patient underwent an image-guided core-needle biopsy of the retroperitoneal mass. Histopathologic examination revealed viable seminoma with focal tumor necrosis (Figure 3). Immunohistochemical staining showed nuclear expression of OCT4 and SALL4 and expression of CD117, whereas glypican-3 and CD30 were negative, which supported the diagnosis of seminoma. The sampled tissue showed no morphologic or immunohistochemical evidence of yolk sac tumor or embryonal carcinoma (Figure 4).

**Figure 3:**
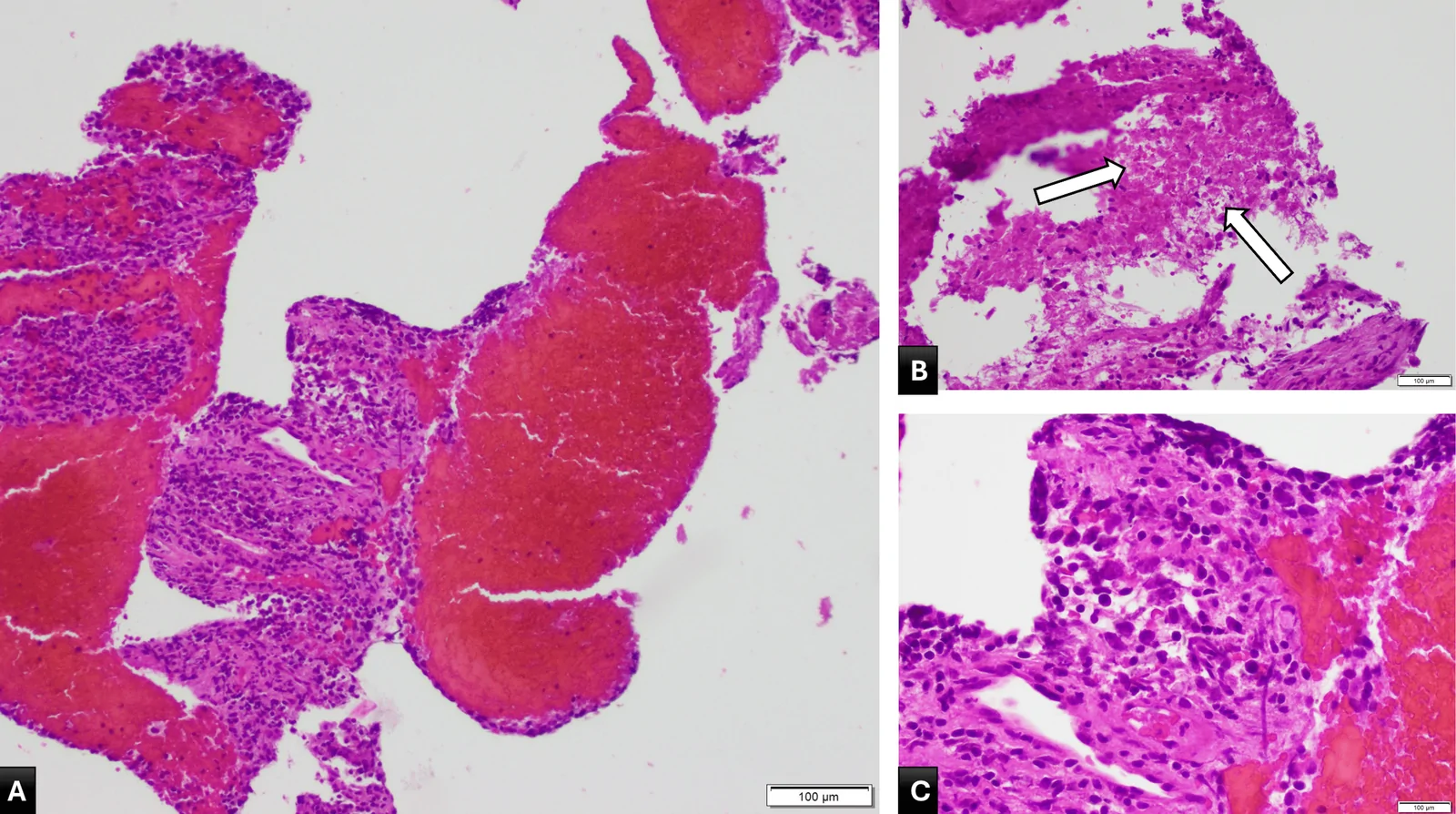
Histopathologic features of seminoma with focal tumor necrosis Hematoxylin and eosin-stained sections of the retroperitoneal mass core biopsy demonstrate viable seminoma with associated hemorrhage (A and C). A focal area of tumor necrosis is present in panel B (arrows). Scale bars represent 100 μm

**Figure 4:**
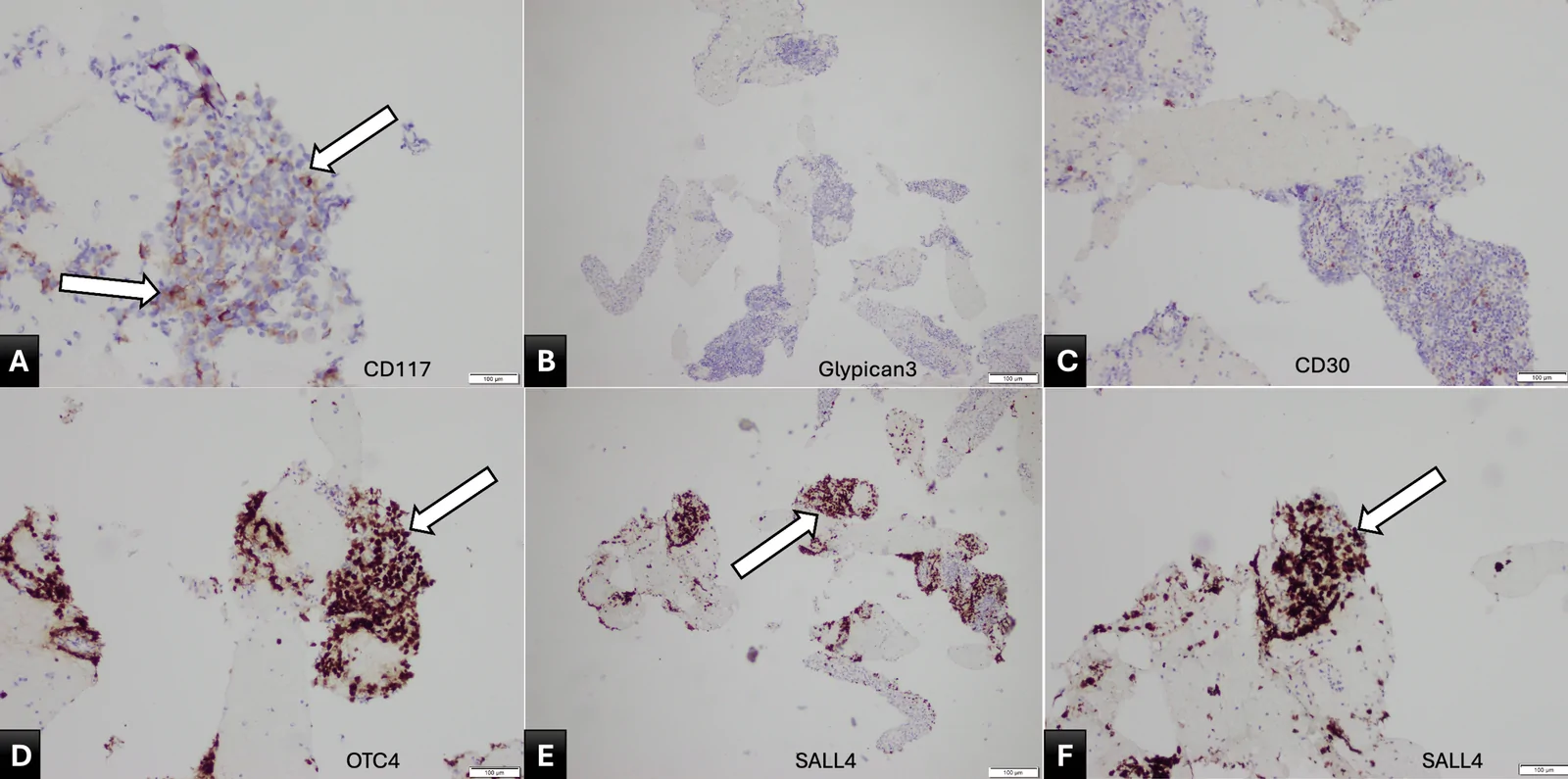
Immunohistochemistry Immunohistochemical staining of the retroperitoneal mass core biopsy demonstrates tumor-cell positivity for CD117 (A), OCT4 (D), and SALL4 (E and F). The tumor cells are negative for glypican-3 (B) and CD30 (C). Arrows indicate immunoreactive tumor cells. This immunohistochemical profile supports the diagnosis of seminoma. No morphologic or immunohistochemical evidence of yolk sac tumor or embryonal carcinoma is identified in the sampled tissue. Scale bars represent 100 μm

Multidisciplinary board discussion

Medical oncology, urology, surgical oncology, interventional radiology, and internal medicine teams participated in the evaluation. Treatment planning took into account the original mixed histology, the lack of prior systemic therapy, the bulky retroperitoneal recurrence, and its relationship to the IVC and right iliac vessels. Although the biopsy of the recurrence showed seminoma, the original embryonal carcinoma component remained relevant because the limited sampling could not exclude nonseminomatous elements elsewhere in the mass. Upfront resection was not favored because of the size of the mass, its vascular relationships, and the anticipated surgical morbidity. The team recommended curative-intent cisplatin-based chemotherapy, followed by assessment of any residual disease.

The nodal mass met the clinical N3 size criterion, and staging imaging showed no definite distant metastases [6]. However, a definitive overall stage and International Germ Cell Cancer Collaborative Group (IGCCCG) risk category at treatment initiation could not be confirmed with confidence from the available documentation. The initial oncology assessment regarded the disease as intermediate-risk nonseminomatous disease on the basis of the original mixed histology and elevated admission LDH, and four cycles of bleomycin, etoposide, and cisplatin (BEP) were initially proposed. Subsequent LDH levels declined before and around treatment initiation, including a value of 371 U/L on August 13. The available records did not establish how these later results had been incorporated into the final risk classification or whether the proposed number of cycles was revised.

Diagnosis and management

BEP chemotherapy was started on August 13, 2026. The planned schedule consisted of bleomycin on days 1, 8, and 15, with etoposide at 100 mg/m² and cisplatin at 20 mg/m² on days 1 to 5. Intravenous hydration was given during cisplatin administration, and electrolytes were monitored. Blood counts, renal function, magnesium, and liver chemistry were also monitored. Supportive treatment included ondansetron, dexamethasone, and olanzapine as antiemetic prophylaxis and enoxaparin as venous thromboembolism prophylaxis. Hiccups associated with treatment were managed with baclofen. Outpatient bleomycin administration and further chemotherapy cycles were planned.

Clinical outcome

During the documented early treatment period, his abdominal pain improved, and the patient remained afebrile and hemodynamically stable. Serial examinations showed a soft, nontender abdomen and no lower-extremity edema. Hiccups were the principal early adverse effect documented. These findings reflected symptomatic improvement and initial treatment tolerance, but they did not establish an objective oncologic response.

On August 20, 2026, the patient underwent CT pulmonary angiography to evaluate nausea, vomiting, and shortness of breath. No pulmonary embolism was identified. The previously documented pulmonary micronodules were unchanged, and no thoracic lymphadenopathy was seen. Subtle bilateral ground-glass opacities were interpreted as possibly representing mild pulmonary edema.

At the end of the available follow-up, curative-intent chemotherapy was to be continued. Information on completion of the full chemotherapy course, subsequent tumor-marker normalization, interval change in retroperitoneal mass size, postchemotherapy residual-mass management, and longer-term oncologic outcomes was not available. The principal diagnostic and treatment milestones are summarized in Table 2.

**Table 2:** Clinical timeline of initial diagnosis, retrospective imaging findings, recurrence, and initial management The 2.1-cm focus was recognized retrospectively during comparison with the 2026 examination; the available records did not establish that it had been identified as suspicious in 2022 AFP: alpha-fetoprotein; BEP: bleomycin, etoposide, and cisplatin; β-hCG: beta-human chorionic gonadotropin; CT: computed tomography; IVC: inferior vena cava; LDH: lactate dehydrogenase; NSGCT: nonseminomatous germ cell tumor

Time point	Clinical event and findings	Management and clinical significance
August 2021	Right radical orchiectomy demonstrated a 6.5-cm mixed germ cell tumor composed of seminoma (80%) and embryonal carcinoma (20%), with lymphovascular invasion. The tumor was confined to the testis, and margins were negative	No adjuvant chemotherapy or retroperitoneal lymph-node dissection was performed; surveillance was selected
June 2022	During the 2026 comparison, a 2.1-cm retroperitoneal focus was retrospectively identified on the June 2022 CT at the site of the later mass. The available 2026 report summarized the earlier examination as showing no acute findings	The available record did not show that the focus was recognized as suspicious or prompted oncologic reassessment. No intervening imaging was available
July 2026	The patient presented with abdominal pain radiating to the back and intermittent constipation. CT showed a 14.1-cm retroperitoneal mass with near-complete IVC effacement. LDH was elevated, β-hCG minimally elevated, and AFP normal	Direct comparison with the earlier study supported progressive nodal recurrence. Tissue sampling and multidisciplinary evaluation were pursued
August 2026	Core biopsy demonstrated seminoma with focal necrosis. Tumor cells expressed OCT4, SALL4, and CD117 and were negative for glypican-3 and CD30	Seminoma was established in the sampled tissue, but unsampled nonseminomatous disease could not be excluded. Available findings supported cN3 cM0 S2, stage IIIB disease
August 2026	Curative-intent BEP was initiated under an intermediate-risk NSGCT framework. The patient completed the inpatient portion of cycle 1 with improved pain and no major acute complications	Four cycles were planned. Completion of treatment, marker response, restaging, and long-term outcome were unavailable

## Discussion

The central diagnostic issue in this case is apparent histologic discordance rather than proven histologic transformation. The orchiectomy specimen was already predominantly seminomatous but also contained a clinically meaningful embryonal carcinoma component, whereas the core biopsy of the recurrence sampled only seminoma. Mixed germ cell tumors show substantial spatial heterogeneity, and a needle core from a 14.1-cm heterogeneous mass cannot establish uniform histology throughout the unsampled regions [2,7,8]. In a study of 118 patients with matched orchiectomy and retroperitoneal lymph-node dissection specimens, Spratt et al. found that agreement between the two sites was lower, and the occurrence of germ cell tumor components more random, than within orchiectomy specimens [8]. Che et al. reported discordant retroperitoneal and thoracic residual histology in 23% of 50 patients with metastatic nonseminomatous germ cell tumors after chemotherapy [9]. Although that postchemotherapy, multisite population differs from the present chemotherapy-naïve recurrence, the finding reinforces that the histology of one specimen or anatomical site cannot always be extrapolated to the entire disease burden.

The biopsy established viable seminoma within the sampled cores. Nuclear OCT4 and SALL4 expression and CD117 positivity, together with negative glypican-3 and CD30 staining, supported seminomatous differentiation, and no morphologic or immunohistochemical evidence of yolk sac tumor or embryonal carcinoma was found in the sampled tissue. However, these findings did not exclude a geographically separate nonseminomatous component elsewhere in the mass. A normal AFP level and a minimally elevated β-hCG level were compatible with seminoma but were not independently diagnostic because conventional markers do not identify every nonseminomatous tumor [10]. Because the patient had received no chemotherapy before recurrence, treatment-driven histologic selection could not explain the biopsy finding; pre-existing heterogeneity of components and limited sampling were more plausible than biological transformation.

The radiographic history also requires careful interpretation. During the 2026 comparison, a 2.1-cm focus was retrospectively identified on the June 2022 study at the site of the subsequent mass. The available 2026 report summarized the earlier examination as showing no acute findings, and the record did not establish that the focus had been recognized as suspicious, communicated as a possible recurrence, or intentionally observed without treatment. Accordingly, the case should be described as progressive nodal recurrence with incomplete documentation of the intervening surveillance rather than progression of a knowingly untreated lesion. Surveillance remains an accepted strategy after orchiectomy, but lymphovascular invasion and an embryonal carcinoma component increase relapse risk, and safe surveillance requires adherence to scheduled clinical review, tumor-marker testing, and cross-sectional imaging [3,5,6,11,12]. New abdominal or back pain or gastrointestinal symptoms in a patient with a prior testicular germ-cell tumor should prompt timely recurrence evaluation and direct comparison with all available earlier imaging, even when AFP is normal and β-hCG is only minimally elevated [1,6].

The recurrence can be classified more explicitly using the available data. A regional nodal mass greater than 5 cm meets the cN3 criterion. Because the pulmonary micronodules were stable and were not considered definite metastases, the available imaging supported a cM0 classification. The pretreatment LDH level of 630 U/L was 2.25 times the laboratory upper limit of normal and met the S2 criterion. The resulting classification was therefore consistent with pT2 cN3 cM0 S2 disease, which corresponds to AJCC stage IIIB [3,6]. Because any embryonal carcinoma component places a mixed primary tumor within the nonseminomatous category, the testicular primary, the absence of nonpulmonary visceral metastases, and the S2 LDH level supported an intermediate-risk nonseminomatous germ-cell tumor framework [3,6]. This classification explains why four cycles of BEP were considered. In contrast, if the entire recurrence could have been established as seminoma, the absence of nonpulmonary visceral metastases would generally indicate good-risk disease; however, the limited core biopsy was insufficient to justify that assumption.

Near-complete IVC effacement added procedural and thromboembolic complexity even though the patient had no peripheral edema. Contemporary guidance favors cisplatin-based systemic therapy for bulky stage IIC or stage III germ-cell tumor, with the regimen and number of cycles determined by histology and IGCCCG risk classification [3,6,13]. Upfront resection has little established role in bulky advanced seminoma and was particularly unfavorable for a mass involving major vessels. In a two-institution series of postchemotherapy retroperitoneal lymph-node dissection, 17% of patients with complete imaging and clinical data required great-vessel resection or reconstruction [14]. In a separate 295-case cohort, 20% required an additional surgical procedure and 9.8% experienced major early complications [15]. Although these series do not directly represent an untreated seminomatous mass, they illustrate the technical demands of operating on vessel-involving retroperitoneal disease.

Multidisciplinary planning was therefore central to treatment selection. Medical oncology integrated the original mixed pathology, the current biopsy, tumor markers, and risk classification; urology and surgical oncology assessed the feasibility and morbidity of resection; interventional radiology obtained tissue; and vascular surgical expertise would be required if a subsequent operation involved the IVC or iliac vessels. This approach supported systemic therapy first while preserving surgery as a response-dependent option.

Management of any postchemotherapy residual mass will remain challenging because seminoma and nonseminoma algorithms differ. In seminoma, a residual mass larger than 3 cm is generally evaluated with delayed fluorodeoxyglucose positron-emission tomography (FDG-PET) and serial imaging, whereas in nonseminoma, resection is generally recommended for a residual mass larger than 1 cm when serum markers normalize appropriately [3,6,13]. Histologic differences between residual sites further argue against allowing one core biopsy to determine all later management [9]. Because the original tumor contained embryonal carcinoma, any residual mass should be reviewed at an experienced multidisciplinary center using both seminoma and nonseminoma considerations.

The patient's early pain improvement and initial treatment tolerance do not establish an oncologic response. Completion of chemotherapy, serial tumor-marker measurements, re-staging imaging, and documentation of residual-mass management are required before treatment effectiveness or long-term prognosis can be assessed. Table 3 summarizes a focused comparison between this case and other cases in the current literature.

**Table 3:** Focused comparison of the present case with the existing literature Evidence summarized from Chavarriaga et al. [1], Iczkowski [2], Oldenburg et al. [3], Zengerling et al. [5], Patrikidou et al. [6], Lobo et al. [7], Spratt et al. [8], Che et al. [9], Pedrazzoli et al. [10], Rico et al. [11], Ruf et al. [12], Passarelli et al. [13], Johnson et al. [14], and Latarius et al. [15] BEP: bleomycin, etoposide, and cisplatin; IVC: inferior vena cava; LDH: lactate dehydrogenase; NSGCT: nonseminomatous germ cell tumor; RPLND: retroperitoneal lymph node dissection; ULN: upper limit of normal

Clinical issue	Evidence from the literature	Application to the present case
Apparent histologic discordance	Germ cell tumor components may differ between orchiectomy and retroperitoneal specimens, and residual sites may show discordant histology [2,7-9]	The core biopsy proved seminoma in the sampled tissue but did not establish transformation or uniformly pure seminoma throughout the mass
Surveillance and warning symptoms	Lymphovascular invasion increases relapse risk, while surveillance requires structured imaging, serum markers, and clinical review [3,5,6,11,12]. Retroperitoneal recurrence may cause abdominal or back pain and gastrointestinal symptoms [1,6]	The 2.1-cm focus was recognized retrospectively, and no intervening imaging was available. New abdominal and back symptoms appropriately prompted renewed imaging and comparison with the earlier study
Stage, risk, and systemic therapy	A nodal mass greater than 5 cm is cN3; S2 disease includes LDH 1.5–10 times ULN. Intermediate-risk NSGCT is treated with four cycles of BEP [3,6,13]	The available findings supported cN3 cM0 S2, stage IIIB disease and an intermediate-risk NSGCT treatment framework because the original tumor contained embryonal carcinoma
IVC involvement and surgery	Major-vessel resection or reconstruction may be required during complex postchemotherapy RPLND, and outcomes favor specialized multidisciplinary centers [14,15]	Near-complete IVC effacement made upfront surgery unfavorable. Systemic therapy was selected first, with vascular surgical planning reserved for a persistent resectable mass
Residual-mass management	Seminoma and NSGCT use different postchemotherapy residual-mass algorithms [3,6,13]	Any persistent mass must be assessed using the original mixed pathology, biopsy limitations, markers, and treatment response rather than as unequivocally pure seminoma

What we learned from this case 

This case reinforced that surveillance after orchiectomy is an active management strategy that requires reliable longitudinal clinical and radiographic reassessment. New abdominal or back pain, gastrointestinal symptoms, or signs of venous obstruction in a patient with a history of testicular germ-cell tumor should prompt timely evaluation for retroperitoneal recurrence, even when conventional tumor markers are normal or only minimally elevated. Direct comparison with all available earlier imaging may reveal progressive disease that was not apparent on the current examination alone.

A second lesson concerned the limits of tissue sampling. The identification of seminoma in a core biopsy established its presence in the sampled tissue but did not prove that the entire heterogeneous mass was pure seminoma. The apparent discordance with the original mixed tumor required integrating the orchiectomy specimen, the biopsy of the recurrence, serum markers, and imaging, rather than allowing one limited specimen to define the complete disease burden.

Finally, near-complete IVC effacement materially changed treatment planning despite the absence of peripheral edema. Early multidisciplinary evaluation supported systemic therapy before a potentially hazardous operation and allowed the need for future surgery to depend on treatment response. Similar cases require a predefined plan that covers marker assessment, restaging imaging, residual-mass management, and vascular surgical involvement if resection becomes necessary.

Limitations

This report is limited by its single-patient design and cannot establish the frequency or biological mechanism of apparent histologic discordance between a mixed primary germ cell tumor and a recurrent-site biopsy. During the 2026 comparison, a 2.1-cm focus was retrospectively identified on the June 2022 examination; however, the available records did not establish whether the focus had been recognized as suspicious at that time, communicated to the treating team, or evaluated outside the available health system. No intervening cross-sectional imaging was available, preventing determination of the lesion’s growth rate or the precise timing of progression.

The recurrent mass was evaluated by core-needle biopsy rather than complete excision. Given the size and heterogeneous radiographic appearance of the mass, sampling error remains possible, and an unsampled embryonal carcinoma or other nonseminomatous component cannot be excluded. Centralized comparative review or molecular analysis of the original and recurrent specimens was not available. The pulmonary micronodules also remained indeterminate, although they were stable and lacked definite features of metastatic disease.

The stage and risk classification were reconstructed from the available pretreatment laboratory and imaging data. The oncology record documented consideration of an intermediate-risk nonseminomatous germ cell tumor with S2 status based on lactate dehydrogenase, but an immediate prechemotherapy marker panel was not available for independent confirmation. Most importantly, follow-up ended during the initial chemotherapy cycle. Completion of treatment, serial marker kinetics, radiographic response, residual-mass management, recurrence-free survival, and long-term toxicities were unavailable. Consequently, this report cannot establish treatment effectiveness or long-term prognosis.

## Conclusions

This case report demonstrates a progressive, bulky retroperitoneal recurrence with near-complete IVC effacement after orchiectomy for a mixed seminoma-embryonal carcinoma, while a limited recurrent-site core biopsy sampled only seminoma. This finding represents apparent histologic discordance rather than proven transformation or definitively pure seminoma. The principal clinical lesson is that recurrent-site biopsy findings must be integrated with the original pathology, pretreatment serum markers, serial imaging, and anatomical relationships when selecting therapy. New abdominal or back symptoms should prompt timely imaging and comparison with prior studies. Multidisciplinary assessment supported cisplatin-based chemotherapy before a potentially hazardous vessel-involving surgical procedure; however, the limited follow-up prevents conclusions regarding the definitive treatment response.

## References

[1] Chavarriaga J, Nappi L, Papachristofilou A, Conduit C, Hamilton RJ (2025). Testicular cancer. Lancet.

[2] Iczkowski KA (2023). Germ cell neoplasms of the testis: update for 2022. Semin Diagn Pathol.

[3] Oldenburg J, Berney DM, Bokemeyer C (2022). Testicular seminoma and non-seminoma: ESMO-EURACAN Clinical Practice Guideline for diagnosis, treatment and follow-up. Ann Oncol.

[4] Padayachee J, Clark R, Warde P, Hamilton RJ (2022). Management of stage I testicular cancer. Curr Opin Urol.

[5] Zengerling F, Beyersdorff D, Busch J (2022). Prognostic factors in patients with clinical stage I nonseminoma-beyond lymphovascular invasion: a systematic review. World J Urol.

[6] Patrikidou A, Cazzaniga W, Berney D (2023). European Association of Urology guidelines on testicular cancer: 2023 update. Eur Urol.

[7] Lobo J, Jerónimo C, Henrique R (2021). Morphological and molecular heterogeneity in testicular germ cell tumors: implications for dedicated investigations. Virchows Arch.

[8] Spratt DE, Suresh K, Osawa T (2018). Detailed pathologic analysis on the co-occurrence of non-seminomatous germ cell tumor subtypes in matched orchiectomy and retroperitoneal lymph node dissections. Med Oncol.

[9] Che Y, Wöltjen C, Lusch A (2024). Discordance of retroperitoneal and thoracic histologic findings in patients with metastatic germ cell tumors at postchemotherapy residual tumor resection. World J Surg Oncol.

[10] Pedrazzoli P, Rosti G, Soresini E, Ciani S, Secondino S (2021). Serum tumour markers in germ cell tumours: from diagnosis to cure. Crit Rev Oncol Hematol.

[11] Rico L, Blas L, Vitagliano G (2021). Active surveillance for testicular tumors: adherence and safety (Article in Spanish). Arch Esp Urol.

[12] Ruf CG, Schmidt S, Kliesch S (2022). Testicular germ cell tumours' clinical stage I: comparison of surveillance with adjuvant treatment strategies regarding recurrence rates and overall survival-a systematic review. World J Urol.

[13] Passarelli R, Pfail JL, Jang TL (2024). Contemporary surgical management of testicular seminoma. Transl Cancer Res.

[14] Johnson SC, Smith ZL, Nottingham C (2019). Clinical and radiographic predictors of great vessel resection or reconstruction during retroperitoneal lymph node dissection for testicular cancer. Urology.

[15] Latarius S, Leike S, Erb H, Putz J, Borkowetz A, Thomas C, Baunacke M (2023). Retroperitoneal lymph node dissection for testicular cancer is a demanding procedure: detailed real-life data of complications and additional surgical procedures in 295 cases. World J Urol.

